# *De novo* transcriptome sequencing and sequence analysis of the malaria vector *Anopheles sinensis* (Diptera: Culicidae)

**DOI:** 10.1186/1756-3305-7-314

**Published:** 2014-07-07

**Authors:** Bin Chen, Yu-Juan Zhang, Zhengbo He, Wanshun Li, Fengling Si, Yao Tang, Qiyi He, Liang Qiao, Zhentian Yan, Wenbo Fu, Yanfei Che

**Affiliations:** 1 Institute of Entomology and Molecular Biology, College of Life Sciences, Chongqing Normal University, Chongqing, P R, China; 2 BGI-Shenzhen, Shenzhen, P R, China

**Keywords:** Anopheles sinensis, Transcriptome, RNA-Seq, Codon usage bias, Simple sequence repeat, Malaria, Vector control

## Abstract

**Background:**

*Anopheles sinensis* is the major malaria vector in China and Southeast Asia. Vector control is one of the most effective measures to prevent malaria transmission. However, there is little transcriptome information available for the malaria vector. To better understand the biological basis of malaria transmission and to develop novel and effective means of vector control, there is a need to build a transcriptome dataset for functional genomics analysis by large-scale RNA sequencing (RNA-seq).

**Methods:**

To provide a more comprehensive and complete transcriptome of *An. sinensis,* eggs, larvae, pupae, male adults and female adults RNA were pooled together for cDNA preparation, sequenced using the Illumina paired-end sequencing technology and assembled into unigenes. These unigenes were then analyzed in their genome mapping, functional annotation, homology, codon usage bias and simple sequence repeats (SSRs).

**Results:**

Approximately 51.6 million clean reads were obtained, trimmed, and assembled into 38,504 unigenes with an average length of 571 bp, an N50 of 711 bp, and an average GC content 51.26%. Among them, 98.4% of unigenes could be mapped onto the reference genome, and 69% of unigenes could be annotated with known biological functions. Homology analysis identified certain numbers of *An. sinensis* unigenes that showed homology or being putative 1:1 orthologues with genomes of other Dipteran species. Codon usage bias was analyzed and 1,904 SSRs were detected, which will provide effective molecular markers for the population genetics of this species.

**Conclusions:**

Our data and analysis provide the most comprehensive transcriptomic resource and characteristics currently available for *An. sinensis*, and will facilitate genetic, genomic studies, and further vector control of *An. sinensis.*

## Background

The *Anopheles sinensis* Wiedemann is a major malaria vector in China and other southeastern Asia countries with wide distribution from Afghanistan to northern China, Korea, Japan, Taiwan, and southward into western Indonesia [1-5]. An investigation of mosquito abundance in Yancheng, Jiangsu, China showed that *An. sinensis* accounted for 60.6% of the total number collected in the Rare Birds National Nature Reserve [6]. From the early 21^st^ century, malaria re-emerged in central China. A total of 64,178 malaria cases and 52,082 suspected cases with 38 deaths were reported in 917 counties of 23 Provinces in 2006, 66.4% of which came from Huang-Huai Plain [7]. Malaria outbreaks and re-emergences appeared only in the areas with *An. sinensis* in recent years, and resulted from the extensive distribution, high population density and increasing vectorial capacity of *An. sinensis* in China [8-11]. In addition to its principal role in the transmission of malaria, this mosquito also plays a role in the transmission of filarial nematodes [12,13]. Mosquito vector control is one of most effective measures to prevent malaria transmission and relies primarily on the use of pyrethroids through insecticide-impregnated bednets and indoor residual spraying at present [14]. However, high levels of resistance to pyrethroids have been reported in *An. sinensis* from China and Korea [15-17]. To better understand the biological basis of *An. sinensis*, especially the molecular mechanisms of malaria transmission and insecticide resistance, there is a need to explore the transcriptomic biology of this mosquito species. Earlier, Jo *et al*. (2011) sequenced and analyzed two cDNA libraries from *An. sinensis* challenged with and without actinomycin-D; however, the overall transcriptomic information of *An. sinensis* is still very limited [18]. As far as March 2013, only 573 nucleotide sequences, 5 expressed sequence tags (ESTs), and 64 protein sequences from *An. sinensis* have been available in GenBank.

Advances in next-generation sequencing (NGS) and assembly algorithm rapidly promote the development of transcriptome sequencing (RNA-seq), which can reconstruct the entire transcriptome in a selected species of interest and generate quantitative expression scores for each transcript [19]. This transcriptome analysis will likely replace large-scale microarray approaches [20,21], as it’s lower cost, greater sequence yield, and higher sensitivity in detection of low abundant and novel transcripts allows the measurement of transcriptome composition to address the quantitative survey of RNA expression patterns in comparative genomic-levels [19] and develop molecular markers [22]. In the past few years, RNA-seq has been used for a number of genomes investigated, covering bacteria, Archaea and lower eukaryotes to higher eukaryotes. It is particularly effective when a reference genome is not available [23]. NGS transcriptome sequencing has also been successfully applied to mosquito species *Anopheles gambiae*[11,24], *Aedies aegypti*[25,26], *Culex quinquefasciatus*[27], *Anopheles funestus*[28] and *Anopheles albimanus*[29], which clearly demonstrated its utility for functional and evolutionary studies [30,31].

The study aims to construct a reference transcriptome of *An. sinensis* sampled from different developmental stages of egg, larva, pupa, and adult using the Illumina Hiseq2000 sequencing platform, and to present a comprehensive analysis of the *de novo* transcriptome sequencing result. As a result, a total of 38,504 unigenes were assembled and identified, among them 26,650 were annotated. Codon usage bias analysis revealed the characters of codon usage in this species. Moreover, a large number of simple sequence repeats (SSRs) were determined. To our knowledge, this is the first report on the complete transcriptome and characterization of *An. sinensis*, and will facilitate future genetic and genomic studies on this species.

## Methods

### Mosquito culture and total RNA preparation

The colony of *An. sinensis* was reared in the Institute of Entomology and Molecular Biology, Chongqing Normal University, China at 26 ± 1°C with 75 ± 5% relative humidity. Eggs (3 samples at the age of 0, 1 and 2 days of development, respectively), larvae (4 samples at 1^st^, 2^nd^, 3^rd^ and 4th instar, respectively), pupae (6 samples 1, 2 and 3 days old, with both male and female, respectively), male adults (7 samples with 1, 2, 3, 4, 5, 6 and 7 days old), female adults (10 samples 1, 2, 3, 4 and 5 days old, with both before blood meal and after blood meal, respectively) were collected, snap frozen in liquid nitrogen, and then stored at −80°C prior to RNA extraction.

Total RNA was separately extracted using TRIzol Reagent (Invitrogen, Carlsbad, CA, USA) following the manufacturer’s protocol. To eliminate genomic DNA, the RNA samples were treated with RNase-Free DNase I according to manufacturer’s protocol (Qiagen, USA). The RNA integrity was confirmed using the Agilent 2100 Bioanalyzer with a minimum integrity number value of 7. Ten percent, 30%, 20%, 20% and 20% of total RNA quantity separately from eggs, larvae, pupae, male adults and female adults RNA sample were pooled together for cDNA preparation.

### mRNA purification, cDNA synthesis and Illumina sequencing

Beads with Oligo (dT) were used to isolate poly (A) mRNA from total RNA extracted. Fragmentation buffer was added for interrupting mRNA to short fragments. Taking these short fragments as templates, random hexamer-primers were used to synthesize the first-strand cDNA. The second-strand cDNAs were synthesized using buffer, dNTPs, RNaseH and DNA polymerase I. Short fragments were purified with QiaQuick PCR extraction kit (Qiagen, USA) and resolved with EB buffer for end reparation and tailing A. The short fragments were then connected with sequencing adapters. After the agarose gel electrophoresis, the ligated products were purified and amplified with PCR to create the final cDNA library. Finally, the cDNA library was sequenced using Illumina HiSeq™ 2000 in Beijing Genomics Institute (BGI)-Shenzhen, China, according to manufacturer’s instructions.

### *De novo* transcriptome assembly

The raw reads produced from HiSeq™ 2000 were cleaned by removing adapter sequences, low-quality sequences (reads with ‘N’ larger than 5%), and reads with more than 10% Q < 20 bases. The quality reads were assembled into unigenes using short reads assembling program – Trinity with the fellow parameters: min_glue =2, V = 10, edge-thr =0.05, min_kmer_cov = 2, group_pairs_distance = 250 [32]. Reads that contain a certain length of overlap area were first joined to form longer fragments, which are called contigs without gaps. Then the reads were mapped back to contigs; with paired-end reads it was able to detect contigs from the same transcript as well as the distances between these contigs. Next, Trinity connected the contigs, and obtained sequences that no longer could be extended. Such sequences are defined as unigenes. The assembled sequences less than 200 nt were deleted. At last, unigenes were divided into two classes by gene family clustering. One is clusters: several unigenes with similarity higher than 70% were classified into one cluster with the prefix CL. And the other is singletons with the prefix Unigene. To evaluate the quality of unigenes, all unigenes were realigned onto *An. sinensis* reference genome [33] using blat [34] with default setting. Fragment per kb per million reads (FPKM) for each gene of each sample was calculated to show the expression quantity of the gene in the sample, thus avoiding the influence of sequencing length and difference [35]. Each FPKM was log_10_ transformed. The raw reads dataset for the transcriptome of *An. sinensis* was submitted to the NCBI SRA database (http://www.ncbi.nlm.nih.gov/Traces/sra/) with the accession number SRR851144. This Transcriptome Shotgun Assembly project has been deposited at DDBJ/EMBL/GenBank under the accession GBEO00000000. The version described in this paper is the first version, GBEO01000000.

### Functional annotation

The BLASTX search (with E-value < = 1e-5) of each unigene larger than 200 nt was conducted against Nr, Swiss-Prot, KEGG, COG and GO databases, and the BLASTN search (E-value < = 1e-5) against the Nt to predict the function and metabolic pathways of unigenes. The best aligning result was used to decide sequence direction and the coding sequences (CDs) of unigenes, respectively. If results of these protein databases conflicted with each other, a priority order of Nr, Swiss-Prot, KEGG and COG was applied to decide sequence direction of unigenes. ESTScan [36] was used to predict the sequence direction and CDs when unigenes were unaligned to any of the databases. Gene function classifications with gene ontology (GO) annotations of the unigenes, an international standardized gene functional classification system, were determined by Blast2GO program [37]. Based on GO annotation WEGO software [38] was used to display GO functional classification.

### Homology analysis between *An. sinensis* and other Dipteran genomes

To confirm validly assembled sequences and identify sequences with bioinformatic associations with other species, one-directional BLAST and Bi-directional BLAST were conducted between *An. sinensis* transcriptome and other Dipteran translated genomes. Diperant genomes of *An. gambiae* (release 3.6), *Ae. aegypti* (release 1.3) and *Culex quinquefasciatus* (release 1.3) from VectorBase (https://www.vectorbase.org/) and *Drosophila melanogaster* (release r5.47) from Flybase (http://flybase.org/), were downloaded. Peptide sequences equal to or longer than 50 a.a. of these species were employed for the comparisons because 1) amino acid sequences could achieve better hits than nucleotide sequences; 2) using short sequences are not as sensitive as using long sequences, and BLAST parameters need to be changed for short sequences (<50). Firstly, in one-directional BLAST, *An. sinensis* unigenes were used as queries to compare with these four translated genomes using BLASTX (E-value < = 1e-5), and sequences producing significant alignments were calculated and kept for further analysis. Secondly, to conduct Bi-directional BLAST, we then performed a reciprocal search with TBLASTN (E-value < = 1e-5) using these 4 Dipteran genomes as the queries, respectively. The top hit sequence was determined as one-directional’best-hits’ for each query. Pairs of sequences that were each other’s best hit were identified in both directions and regarded as putative 1:1 ortholog genes between *An. sinensis* and other Dipteran genomes. Bi-directional best hit has been widely used to identify the orthologous genes between closely related species and this approach has been demonstrated to perform better than more complex orthology identification algorithms.

To conduct comparative GO classification, the GO annotations for genes in *An. gambiae* genomes were first retrieved from *An. gambiae* genome (release 3.6). The obtained annotations were analyzed by WEGO software (http://wego.genomics.org.cn/cgi-bin/wego/index.pl) [38] to display the distribution of gene functions and the similarity and difference among *An. sinensis* and *An. gambiae.*

### Characterization of ORFs and codon usage

The ORFs in each unigene sequence was predicted by BLAST against protein databases using BLASTX (E-value < = 1e-5) in the following order: Nr, SwissProt, KEGG, COG. Sequences with hits in former databases would not go to next round search against later database. The coding regions were then extracted according to the best BLASTX match with a custom perl script. We analyzed GC content and codon usage bias of ORFs longer than 150 nt by CodonW (http://codonw.sourceforge.net/), and processed the output files of Codonw with a perl script. The correlation analysis was performed using SPSS 13.0 statistics software (http://www-01.ibm.com/software/analytics/spss/).

### SSR Detection

Unigenes of *An. sinensis* longer than 1 kb obtained in this study were subjected to the detection of SSRs using a perl script adapted from Simple Sequence Repeat Identification Tool (SSRIT, http://www.gramene.org/db/markers/ssrtool) [39]. The parameters were designed for identifying perfect di-, tri-, tetra-, penta-, and hexa-nucleotide motifs, with minimum thresholds of six, five, four, four and four repeats, respectively. Mononucleotide repeats were not considered due to the possibility of Illumina homopolymer sequencing problem associated with this technology.

## Results and discussion

### Illumina sequencing and assembly

To obtain a global overview of the *An. sinensis* transcriptome and gene activity at nucleotide resolution, a mixed cDNA sample representing diverse developmental stages of *An. sinensis* was prepared and sequenced using the Illumina Genome Analyzer. The sequencing of each cDNA fragment yielded two independent reads, 90-nt from both ends of the fragment. We obtained a total of 60.87 million raw reads. After the removal of adaptor sequences, ambiguous and low-quality reads, we obtained 51.61 million (84.79% raw reads) of clean reads with 4.64 Giga nucleotides (Gnt), 95.92% Q20, 51.26% GC content, and 0.00% unknown nucleotide ‘N’ (Table 1).

**Table 1:** **Statistics of RNA-seq based sequencing, assembling and functional annotation for *An. sinensis***

**Sequencing results**	**Number of total raw reads**	**60,866,926**
	Number of total clean reads	51,606,364
	Number of total clean nucleotides (nt)	4,644,572,760
	Q20 percentage of total clean reads	95.92%
	GC percentage of total clean nucleotides	51.26%
	N percentage of total clean nucleotides	0.00%
**Assembling results**	Number of unigenes	38,504 (5,372 into distinct clusters; 33,132 singletons)
	Total length (nt) of total unigenes	21,977,286
	Mean length (nt) of total unigenes	571
	N50 (nt) of total unigenes	711
**Annotation**	Unigenes with Nr database	25,456 (66% of 38,504 unigenes)
(E-value < =1e-5)	Unigenes with Nt database	20,554 (53%)
	Unigenes with Swiss-Prot database	17,651 (46%)
	Unigenes with KEGG database	16,622 (43%), 257 pathways
	Unigenes with COG database	7,204 (19%), 25 functional categories
	Unigenes with GO database	16,588 (43%), 62 subcategories grouped to 3 main categories
	Biological process	27 sub-categories
	Cellular component	17 sub-categories
	Molecular function	18 sub-categories
	Total unigenes annotated	26,650 (69% of 38,504 unigenes)

These clean reads were assembled *de novo* by Trinity, which produced 38,504 unigenes (longer than 200 nt), with an average length of 571 nt and a N50 of 711 nt (Table 1). Out of these unigenes, 5,372 (14.0%) could be classified into distinct clusters, 33,132 (86.0%) were distinct singletons, 14,269 (37.1%) were longer than 500 nt, and 4,921 (12.8%) were longer than 1000 nt. The length distributions of unigenes are shown in Additional file 1.

**Figure 1:**
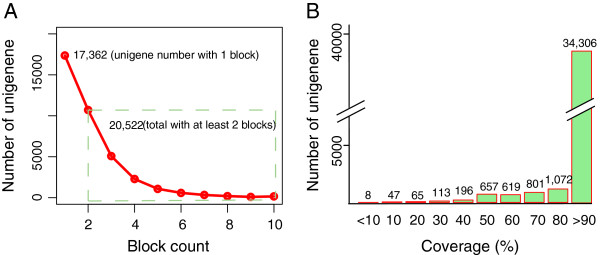
**Distribution of block count (A) and coverage (B) of alignment between *An. sinensis *transcriptome and reference genome.** Coverage is the ratio of match-to-length for each unigene.

The *An. sinensis* reference genome [33] was used to evaluate the assembled transcripts. As a result, about 98.4% (37,884/38,504) of unigenes could be mapped onto the reference genome (Additional file 2). Among them, 17,362 unigenes mapped with 1 block might have 1 exon, or be non-coding RNAs [40] (Figure 1A). The uncertainty could be due to low expression of transcript or assembly issue. The remaining 20,522 unigenes were mapped with at least 2 blocks onto the reference genome, which suggest at least 2 exons. 34,306 (90.6% of 37,884) mapped unigenes could be realigned onto the genome with more than 90% coverage, which supported the quality of the assembled unigenes (Figure 1B).

### Functional annotation

BLASTX search for each unigene sequence was conducted against several protein databases, including NCBI Non-redundant protein database (Nr), NCBI Non-redundant nucleotide database (Nt), Swiss-Prot, the Kyoto Encyclopedia of Genes and Genomes (KEGG), Cluster of Orthologous Groups (COG) databases and Gene Ontology (GO) databases, with an E-value threshold of 1e-5. The results indicated that out of 38,504 unigenes, a total of 25,456 (66%) unigenes were annotated against Nr, 20,554 (53%) against Nt, 17,651 (46%) against Swiss-Prot, 16,622 (43%) against KEGG, 7,204 (19%) against COG, and 16,588 (43%) against GO database (Table 1). Altogether, BLAST searches against Nr, Nt, Swiss-Prot, KEGG, COG and GO databases showed that a total of 26,650 (69% of 38,504 unigenes) identified unigenes could be annotated with known biological functions (Table 1).

**Figure 2:**
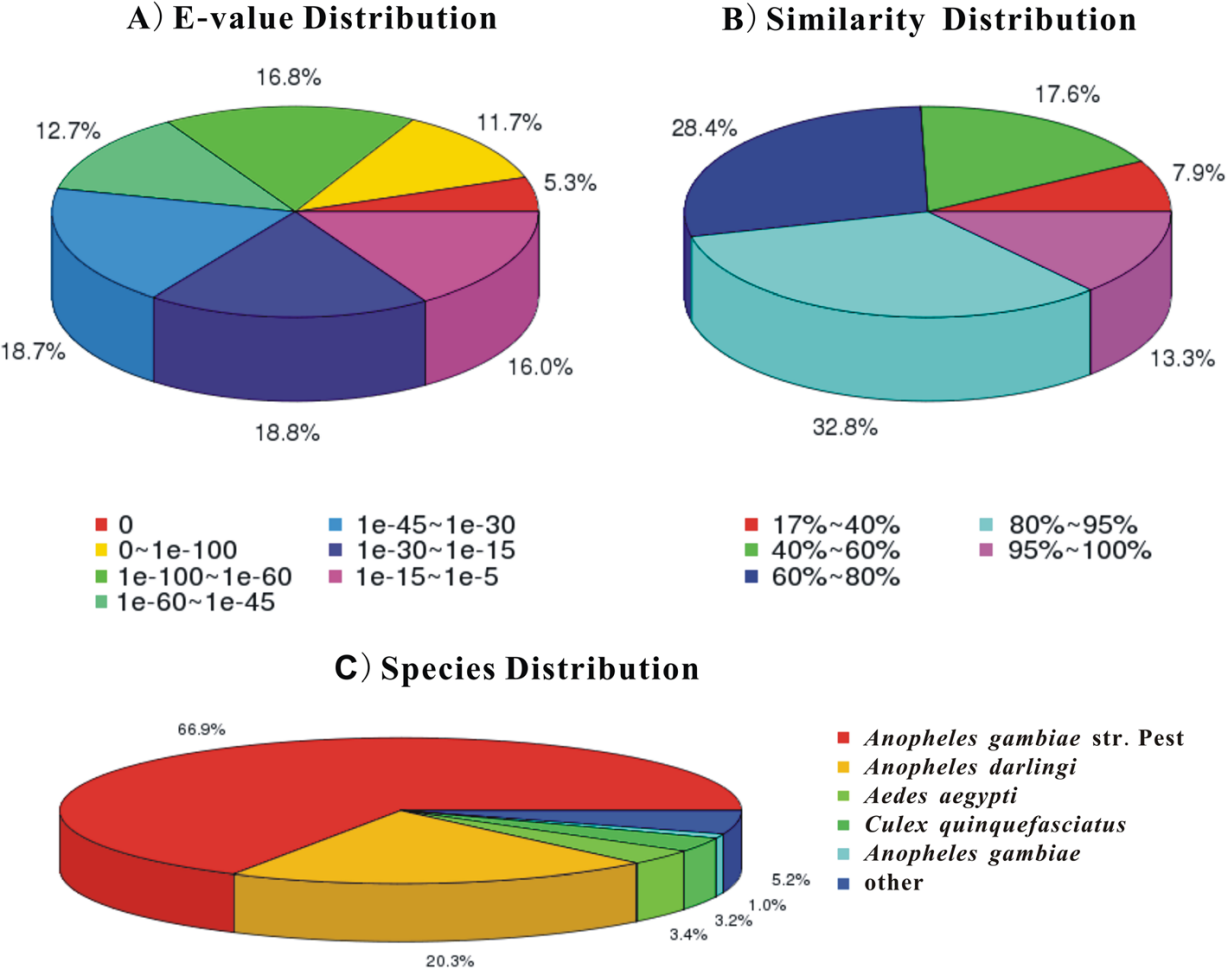
**NR classification of *An. sinensis *unigenes. A)** E-value distribution; **B)** Similarity distribution. **C)** Species distribution.

The E-value distribution, similarity distribution and species distribution against the Nr database were then analyzed (Figure 2). For the E-value distribution of the predicted proteins, 46.5% of the mapped sequences had significant hits with a stringent threshold of less than 1e-45, and 53.5% of the mapped sequences with thresholds from 1e-45 to 1e-5 (Figure 2A). For the similarity distribution of the predicted proteins, 46.1% of the sequences had hits with similarity higher than 80% against the Nr database, and 74.5% of the sequences with similarity higher than 60% (Figure 2B). The species distribution showed that 94.8% of *An. sinensis* unigenes matched to 5 mosquito species, and only 5.2% of unigenes matched other species. Among these 5 mosquito species, 66.9% of the unigenes matched to *An. gambiae* str. PEST, followed by *An. darlingi* with 20.3% matches, *Aedes aegypti* with 3.4% matches, *Culex quinquefasciatus* with 3.2% matches and *An. gambiae* with only 1.0% matches (Figure 2C).

**Figure 3:**
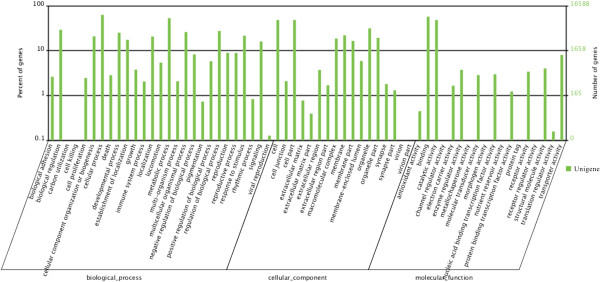
**GO function classification of *An. sinensis *** **unigenes.**

**Figure 4:**
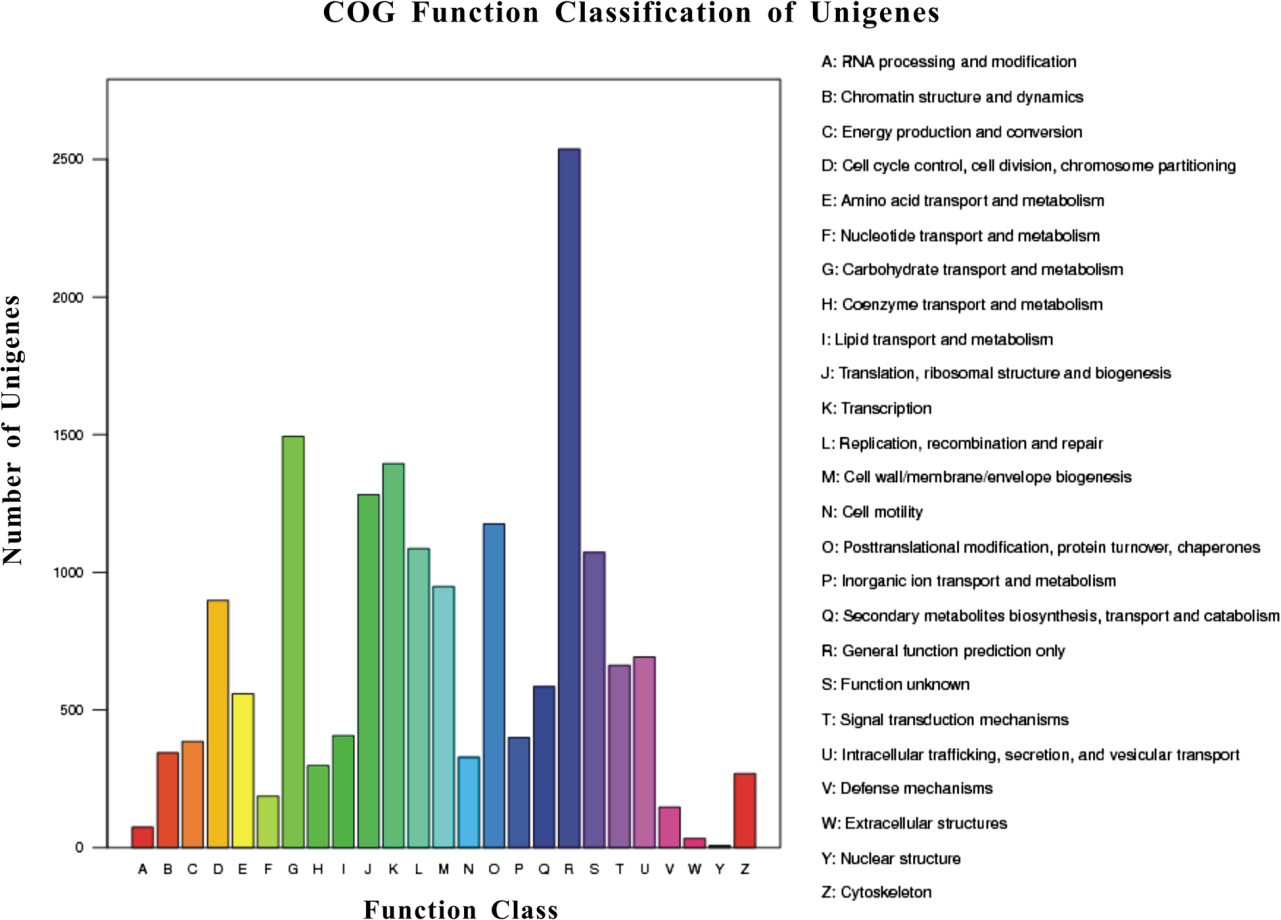
**COG classification of *An. sinensis *** **unigenes.**

Based on GO annotation, 16,588 unigenes were each assigned a gene ontology term and categorized into 62 subcategories belonging to 3 main categories, including biological process (27), cellular component (17), and molecular function (18) (Table 1, Figure 3). In the category of molecular function, the GO term quantity in binding activity and catalytic activity was obviously larger than others, and in the categories of biological processes the subcategories of cellular process and metabolic process appeared somewhat dominant and in cellular component the subcategories of cell and cell part showed a little prevailing (Figure 3). This GO assignment result is similar to the transcriptome earlier sequenced in *Taenia multiceps*, in which binding, cell and metabolic processes were the three largest groups [41]. These GO annotations demonstrated that expressed genes in *An. sinensis* encoded diverse structural, regulatory, metabolic and transporter proteins.As a result of the search of 38,504 unigenes against COG database for orthologous genes, 7,204 unigenes were finally predicted and classified into 25 functional categories. The largest category was “General function prediction only” with 2,537 unigenes (35.2%) that were associated with basic physiological and metabolic functions, followed by “Carbohydrate transport and metabolism” (1,494 unigenes, 20.7%), “Transcription” (1,394, 19.4%), “Translation, ribosomal structure and biogenesis” (1,281, 17.8%) and “Posttranslational modification, protein turnover, chaperones” (1,175, 16.3%). The “RNA processing and modification” (73, 1.0%), “Extracellular structures” (31, 0.4%) and “Nuclear structure” (6, 0.08%) represented the smallest groups of unigenes (Figure 4).

The potential involvement in biological pathways of the assembled unigenes was annotated with corresponding Enzyme commission (EC) numbers and pathways from BLASTX searches against the KEGG database [42]. The KEGG database annotation provided the inner-cell metabolic pathways and functions of gene products of unigenes. Out of 38,504 assembled unigenes, a total of 16,622 unigenes were assigned into 257 KEGG pathways (Table 1). The pathways largely represented by unique sequences were metabolic pathways (2,211 unigenes), RNA transport (675), pathways in cancer (599) and amoebiasis (576). These annotations provided us with a valuable resource for the investigation of specific processes, structures, functions and pathways in *An. sinensis* research.

### Homology analysis between *An. sinensis* and other Dipteran genomes

**Figure 5:**
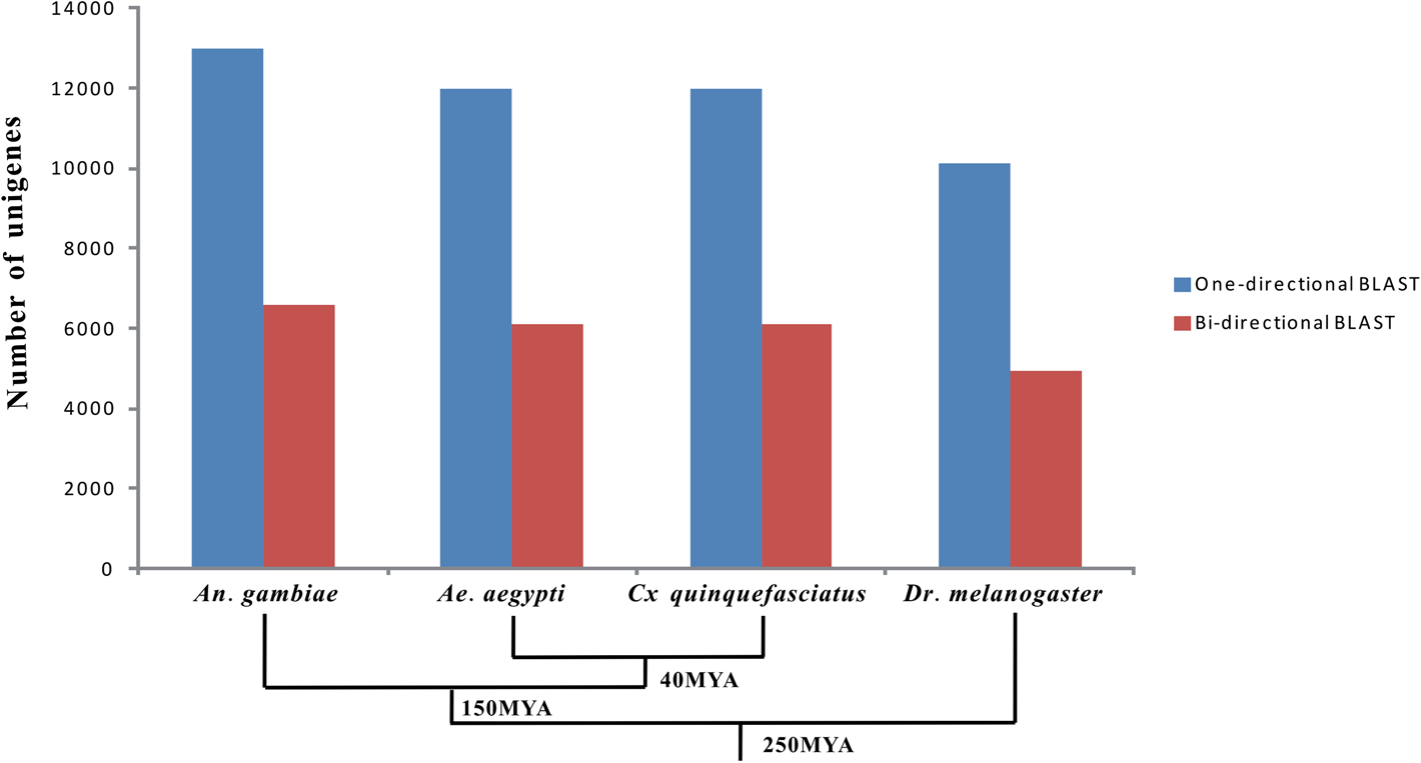
**Homologous gene numbers between *An. sinensis*, and *Ae. aegypti*, *Cx quinquefasciatus *and *Dr. melanogaster *detected by one- and bi-directional BLAST searches.** The numbers decreased with the phylogenetic distance between *An. sinensis* and other Dipteran species (the divergence times were adapted from Grimldi *et al*. [43].

In order to obtain a more detailed understanding of *An. sinensis* biology and reveal a potential evolutionary relationship among Dipteran species, orthologous genes shared between *An. sinensis* and 4 other Dipteran species were compared, including *Drosophila melanogaster*, *An. gambiae, Ae. aegypti* and *Culex quinquefasciatus*. In comparison of the *An. sinensis* unigene sequences to predicted a.a. sequences of other Dipteran species, 12,973 a.a. sequences (90.7% of 14,296) of *An. gambiae* showed a significant similarity to those of *An. sinensis*, followed in decreasing ratio by *Ae. aegypti* with 11,997 (69.1%), *Cx. quinquefasciatus* with 11,990 (63.4%) and *Dr. melanogaster* 10,119 (36.7%) a.a. sequence matches (Table 2 and Figure 5). It was consistent with the expectation that the match ratio of sequences with significant similarity in pairwise comparisons between two species decreased with increasing phylogenetic distance [28]. Bi-directional BLAST was used to identify 1:1 orthologs between *An. sinensis* and other Dipteran species. Putative 6,586, 6,116, 6,084 and 4,919 pairs were identified between *An. sinensis* and *An. gambiae*, *Ae. aegypti*, *Cx. quinquefasciatus*, and *Dr. melanogaster*, respectively (Table 2 and Figure 5). In a previous comparison between *An. funestus* contigs and *An. gambiae* protein sequences, Crawford *et al*. [28] identified 5,434 pairs between *An. funestus* contigs and *An. gambiae* using the standard reciprocal best-hit criteria. The result was comparative with our 1:1 orthologues of 6,586 pairs between *An. sinensis* and *An. gambiae*. The difference might be due to different phylogenetic distance in *Anopheles*, assembling methods and the updated version of *An. gambiae* data.

**Table 2:** **Homology analysis between *An. sinensis *** **and other Dipteran genomes using BLASTX with cut-off E-value of 1E-5**

	**An. gambiae**	***Ae. aegypti***	***Cx quinquefasciatus***	***Dr. melanogaster***
Number of a.a. sequences	14324	17408	19018	27538
Number of sequences with a.a. > 50	14296	17345	18906	27410
Number of one-directional BLAST hits	12973	11997	11990	10119
Number of Bi-directional BLAST hits	6586	6116	6084	4919
Genome sequence version	AgamP3.6	AaegL1.3	CpipJ1.3	r5.47
Source of genome sequence	Vectorbase	Vectorbase	Vectorbase	Flybase

**Figure 6:**
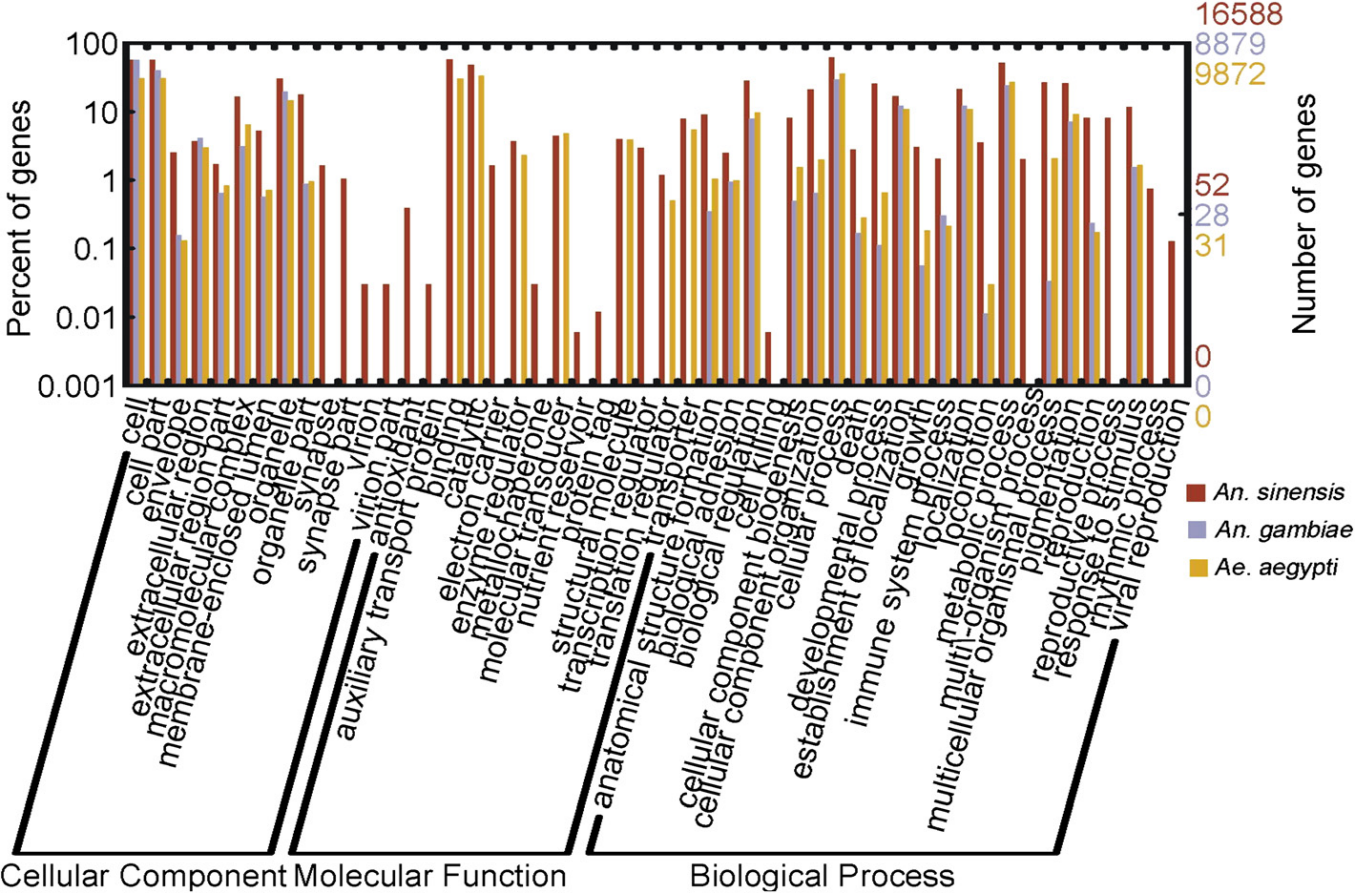
**GO terms similarity distribution among *An. sinensis *** **and *An. gambiae. *** **Bar graph was plotted using a web-based tool, WEGO.**

The GO classification was further used to identify the difference of functional category between *An. sinensis* unigenes and the genes of *An. gambiae*. The result of the comparative analysis of GO terms between *An. sinensis* unigenes and the genes of *An. gambiae* was shown in Figure 6. In total, 16,588 unigenes of *An. sinensis* and 9,872 genes of *An. gambiae* were assigned with one or more GO terms*,* respectively. The major subcategories in the GO classification were shared by the genes of these 2 species of mosquitoes; however, qualitative and quantitative differences existed. The differences might result from the difference both in species and sample source, and further research is needed to elucidate the hypothesis.

### Codon usage bias

Codon usage bias, higher usage frequency of specific condons than other synonymous codons during the translation of genes, can help understand the physiological, biochemical and molecular mechanism as a useful tool in functional genomic research [44]. The extent of codon usage bias may vary within and among species, which can be influenced by various factors, including expression level, GC content, codon position, gene length, environmental stress and population size [45].

In the study, total codon usages were obtained and most frequently, seldom used codons were identified. A total of 24,361 ORFs longer than 150 nt were used in the codon usage bias analyses. A total 4,048,458 counts of codons and the relative synonymous codon usage (RSCU) for these sequences were calculated (Table 3, Additional file 3). Average RSCU values showed that the seven most frequently used codons in *An. sinensis* are: CUG, CCG, UCG, CGC, GUG, ACG and AUC; the seven seldom used codons in *An. sinensis* are: UAU, CCU, ACU, UCU, AGA, AGG and UUA without considering stop codons (Additional file 3). The results are very similar to the results obtained from other Dipteran insects based on the genome-specific frequencies of the codons [46].

**Table 3:** **GC content and codon bias in predicted ORFs of unigenes in *An. sinensis*.**

**Total number of examined ORFs**	**24,361**
% GC of 24,361 ORFs	55.25%
GC3 (% GC at 3rd codon position)	65.40%
Nc (Effective number of condons)	46.71
Total number of codons	4,048,458

The average GC content of 24,361 ORFs was 55.25% and the average GC content of all 38,504 unigenes in *An. sinensis* was 51.26% (Table 3). The unigene GC percentage was similar to the genome GC percentage in *An. gambiae* (55.8%) and *Dr. melanogaster* (53.9%) [46]. The GC content at the third codon position (GC3) was considered to largely decide genome base composition, which varies in different species. Our results showed the average GC3 was 65.40% in *An. sinensis* (Table 3, Additional file 4). To our knowledge, it is the first time the GC3 content in mosquitoes has been reported.

Effective number of codons (Nc), a measure quantifing how far the codon usage of a gene departs from average usage of synonymous codons in study of the state of codon usage bias in genes and genomes [47], has been reported in a range from 20 (maximally bias) to 61 (unbiased) [48]. The mean Nc value of *An. sinensis* was calculated to be 46.71 (Table 3), a medium value in the range, which suggested a medium extent of codon preference in *An. sinensis*[49,50]. A plot of Nc versus GC3 (Nc plot) has been widely used to study the codon usage variation among genes in different organisms [51]. If the codon usage variation among the genes was only determined by variation in GC3 content, then the values of Nc would fall on the continuous curve between Nc and GC3. The Nc plot for *An. sinensis* showed that most of the genes fall within a restricted cloud, at GC3 between 0.013 and 1, and Nc values between 21.15 and 61 (Additional file 4 and 5). Most genes had an Nc value lower than the expected value located on the curve, suggesting that the codon usage of a large number of *An. sinensis* genes are not only determined by GC3.

Commonly, highly expressed genes (such as ribosomal protein genes) tend to reflect greater codon bias than lowly expressed genes [52]. A significant positive correlation between codon usage, bias and gene-expression levels determined from microarray data have been demonstrated in *Ae. aegypti* and *An. gambiae*[53]. To test the effect of codon bias on gene expression in *An. sinensis*, we compared the codon bias index (CBI) values with the observed gene expression levels (log_10_ transformed FPKM) for 24,361 ORFs as determined from whole transcriptome data in *An. sinensis*. We observed a significantly positive correlation between the CBI and gene expression levels in *An. sinensis* (Pearson Correlation: r = 0.037, *P* =1.04e-8) (Additional files 4 and 6)*.*

In our study, total codon usages were obtained, and most frequently, seldom used codons were identified. The Nc plot showed that most genes have an Nc value lower than the expected value located on the curve, suggesting that the codon usage of a large number of *An. sinensis* genes are subject to other factors. We also tested the influence of GC3 and expression levels on codon usage bias in *An. sinensis*. A significant correlation between codon usage and expression levels was observed in *An. sinensis* (Additional file 6)*.* Knowledge of the codon usage pattern in *An. sinensis* can provide a basis for understanding the mechanisms of codon usage bias and for selecting appropriate host expression systems to improve the expression of exogenous genes in *An. sinensis* and thus facilitate vector control.

### SSR discovery

To explore SSR profiles in the *An. sinensis* transcriptome, 4,921 unigene sequences longer than 1 kb were searched for SSRs. As a result, 1,223 sequences containing a total of 1,904 and 307 kinds of SSRs were identified, with 681 of the sequences containing more than 1 SSR. The frequency of SSR in *An. sinensis* transcriptome was 1 per 11.5 kilobases (Table 4). The tri-nucleotide repeat motif was the most abundant, accounting for 57.7%, followed by di-nucleotide repeat motif (36.2%), tetra-nucleotide (5.3%), penta-nucleotide (0.5%), and hexa-nucleotide (0.3%) repeat units (Table 5). The largest abundance of tri-nucleotide repeat motifs is consistent with the result from another insect species *Spodoptera exigua*[54]. The frequencies of SSRs with different numbers of tandem repeats were also calculated and shown in Table 5. SSRs with five tandem repeats (occupying 37.3%) were the most common, followed by six tandem repeats (30.0%), seven tandem repeats (15.5%), eight tandem repeats (6.9%), and four tandem repeats (4.3%). A detailed list of SSRs identified was shown in Additional file 7.

**Table 4:** **Features of SSRs identified in the *An. sinensis *** **transcriptome**

**Total number of examined unigenes**	**38,504**
Number of unigenes longer than 1 kb	4,921
Total nucleotides screened (knt)	21,977
Number of unigenes containing SSRs	1,223
Number of identified SSRs	1,904
Kinds of identified SSRs	307
Number of unigenes containing more than 1 SSRs	681
Frequency of SSR in transcriptome	1/11.5Kb

**Table 5:** **Frequency of SSRs in *An. sinensis *** **transcriptome**

**Number of nucleotides**	**Number of motif repeat**									
	**4**	**5**	**6**	**7**	**8**	**9**	**10**	**>10**	**Total**	**%**
Di	-	-	297	168	111	58	24	31	689	36.2
Tri	-	685	263	126	18	1	1	4	1098	57.7
Tetra	70	22	4	1	3	-	-	2	102	5.3
Penta	7	2	1	-	-	-	-	-	10	0.5
Hexa	4	1	-	-	-	-	-	-	5	0.3
Total	81	710	565	295	132	59	25	37	1904	
%	4.3	37.3	30.0	15.5	6.9	3.1	1.3	1.9		

SSRs are valuable genetic markers used in population genetic analysis and genetic mapping [55]. The traditional isolation of genomic DNA-derived SSRs is difficult and costly. However, large-scale transcriptome sequencing programs based on NGS methods produced large amounts of sequence data, from which the isolation and identification of genome-wide and gene-based SSRs become easier and cheaper. More importantly, SSR markers identified from transcriptome sequences can be used as a tool for identifying associations with functional genes, and therefore with phenotypes [56]. The SSR markers derived from transcriptome databases should be of more benefit, because they should mainly occur in the protein-coding regions of annotated unigenes, which represents genes of known or predicted identity and function.

As the majority of SSR markers derived from transcriptome databases should occur in the protein-coding sequences of annotated unigenes, representing genes of known or predicted identity and function. Efficient discovery of SSR loci based on transcriptome data have been demonstrated in many organisms, such as in *Phlebotomus papatasi*[57], *Sp. exigua*[54]. To our knowledge, this is the first work to identify the SSRs from transcriptome database for mosquitoes, although SSRs in *Cx quinquefasciatus*[58], *An. gambiae*[59] and *An. sinensis*[60] were reported earlier from genomic DNA data. 1,904 SSRs reported herein from *An. sinensis* was much over the number 21 earlier reported from the species [60]. The samples for *An. sinensis* transcriptomes were collected for every developmental stage. Therefore, the 1,904 SSRs should have covered most, if not all, of SSRs in the *An. sinensis* transcriptome, which will contribute to the genome mapping of this species as well as of closely related species, and also assist in the mapping and identification of important functional genes.

## Conclusions

*An. sinensis* is a major malaria vector in China and Southeast Asia; however, the lack of transcriptome data has seriously hindered the research of biology, ecology and molecular mechanisms regarding malaria transmission and control of the mosquito species. To establish a transcriptome resource that will facilitate future genomic level studies in this species, we used the Illumina Hiseq2000 sequencing platform for *An. sinensis* transcriptome sequencing, and produced 38,504 assembled unigenes with 25,456 (66%) annotated. This study dramatically increased the CDs number of genes from *An. sinensis*. To our knowledge, our results represented approximately 70-fold more genes than all *An. sinensis* genes deposited in GenBank (as of Jan, 2013). Certain numbers of them showed homology or could be identified as 1:1 orthologues with other Dipteran genomes, which provided us with significances in evolutionary studies of Dipteran insects. Knowledge of the codon usage pattern in *An. sinensis* provides the basis to understand the mechanisms of codon usage bias and would be useful for future transgenic manipulation in mosquitoes. Moreover, the SSR markers identified may serve as potential marker selection in the mapping and identification of disease-related genes and contribute to the genome mapping and mosquito population genetics research. We believe that results obtained from this study will also serve as a useful genomic dataset to accelerate research of disease transmission mechanisms, resistance mechanisms, and functional genomics in *An. sinensis*. It is possible that some of the results we presented might become useful in the future vector control.

## Abbreviations

CBI: Codon bias index; CDs: coding sequences; COG: Cluster of Orthologous Groups databases; FPKM: Fragment per kilobase per million reads; GC3: GC content at the third codon position; GO: Gene ontology; KEGG: The Kyoto Encyclopedia of Genes and Genomes; Nc: Effective number of codons; Nc plot: A plot of Nc versus GC3; NGS: Next-generation sequencing; Nr: NCBI Non-redundant protein database; Nt: NCBI Non-redundant nucleotide database; ORFs: Open reading frames; RNA-seq: Transcriptome sequencing; RSCU: Synonymous codon usage; SSRs: Simple sequence repeats.

## Competing interests

All authors declare that they have no competing interests.

## Authors’ contributions

BC conceived and designed the study, jointly performed data analysis and drafted the manuscript. YJZ jointly contributed to data analysis and manuscript drafting. WL helped in performing dada analysis. ZH, FS, YT, QH, LQ, ZY, WF and YC conducted sample collecting and experiments. All authors read and approved the final manuscript.

## Supplementary Material

Additional file 1 **Length distribution of unigenes on *An. sinensis *** **reference genome.** Click here for file

Additional file 2 **Alignment between *An. sinensis *** **transcriptome and reference genome.** Only longest alignment was kept in the cases that a unigene was returned with multiple blat hits. Click here for file

Additional file 3 **Total codon usage and codon usage bias in *An. sinensis *** **transcriptome.** Click here for file

Additional file 4 **The ID and FPKM of unigenes, and the CBI, Nc and GC3 of ORF in each unigene in *An. sinensis.***
Click here for file

Additional file 5 **A plot of Nc versus GC3 (Nc-plot) for *An. sinensis *** **ORFs. The pink dotted curve represents the expected curve between GC3 and Nc under random codon usage.** Blue dot each indicates a corresponding ORF of each unigene (totally 24,361 unigenes). Click here for file

Additional file 6 **Relationship between CBI (codon bias index) and expression level (Log10 (FPKM)) of all *An. sinensis *** **transcriptome unigenes.** Click here for file

Additional file 7 **SSR identification of *An. sinensis *** **unigenes.** Click here for file
