# Supplementary figures and images for: De novo transcriptome sequencing and sequence analysis of the malaria vector Anopheles sinensis (Diptera: Culicidae)
Source: Parasit Vectors. 2014 Jul 7;7:314. doi: 10.1186/1756-3305-7-314 (PMC4105132; doi:10.1186/1756-3305-7-314)

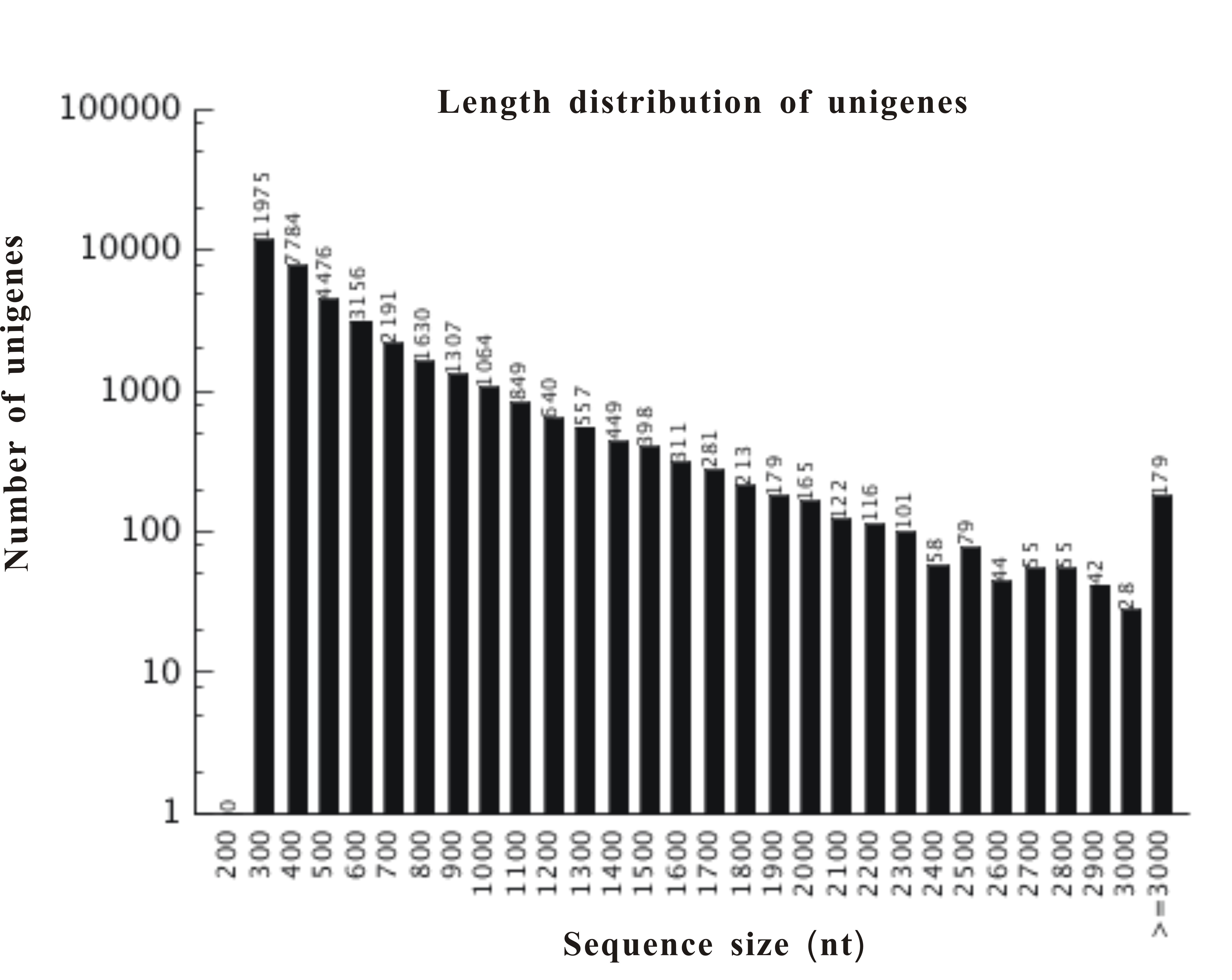

Supplement: Additional file 1 — Length distribution of unigenes on An. sinensis reference genome. [file 1756-3305-7-314-S1.jpeg]

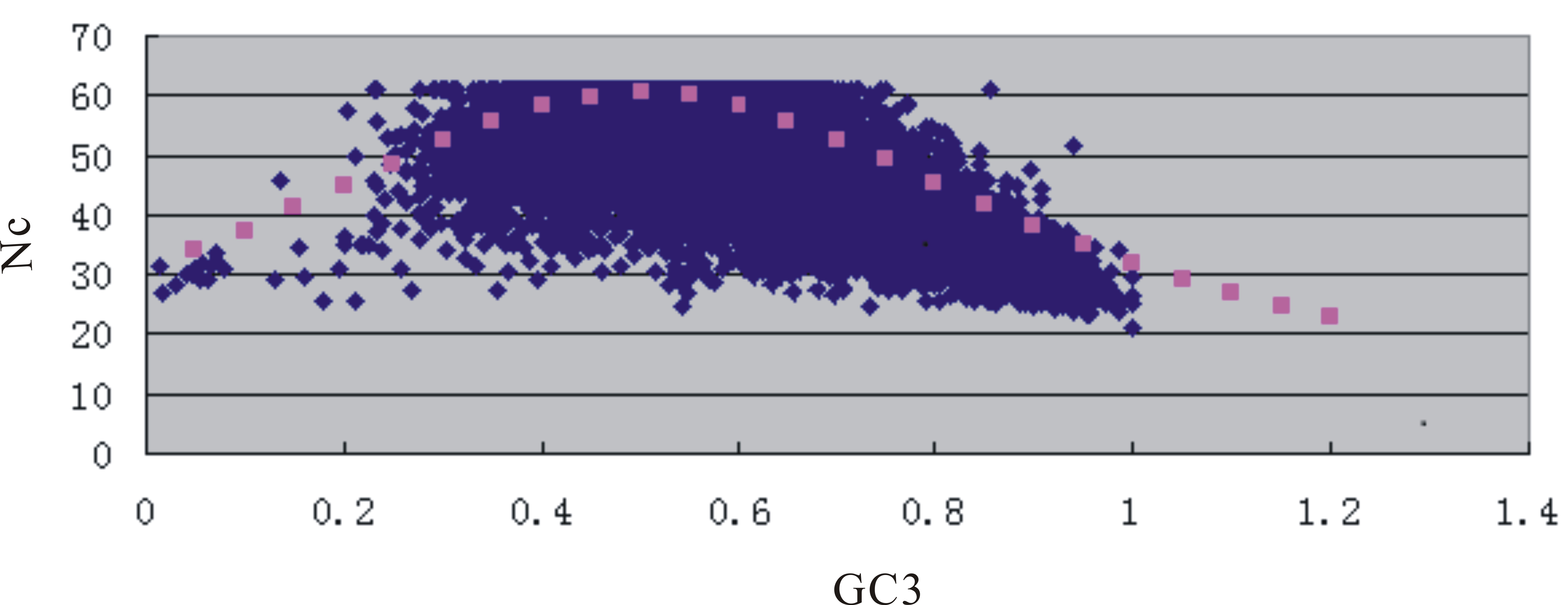

Supplement: Additional file 5 — A plot of Nc versus GC3 (Nc-plot) for An. sinensis ORFs. The pink dotted curve represents the expected curve between GC3 and Nc under random codon usage. Blue dot each indicates a corresponding ORF of each unigene (totally 24,361 unigenes). [file 1756-3305-7-314-S5.jpeg]

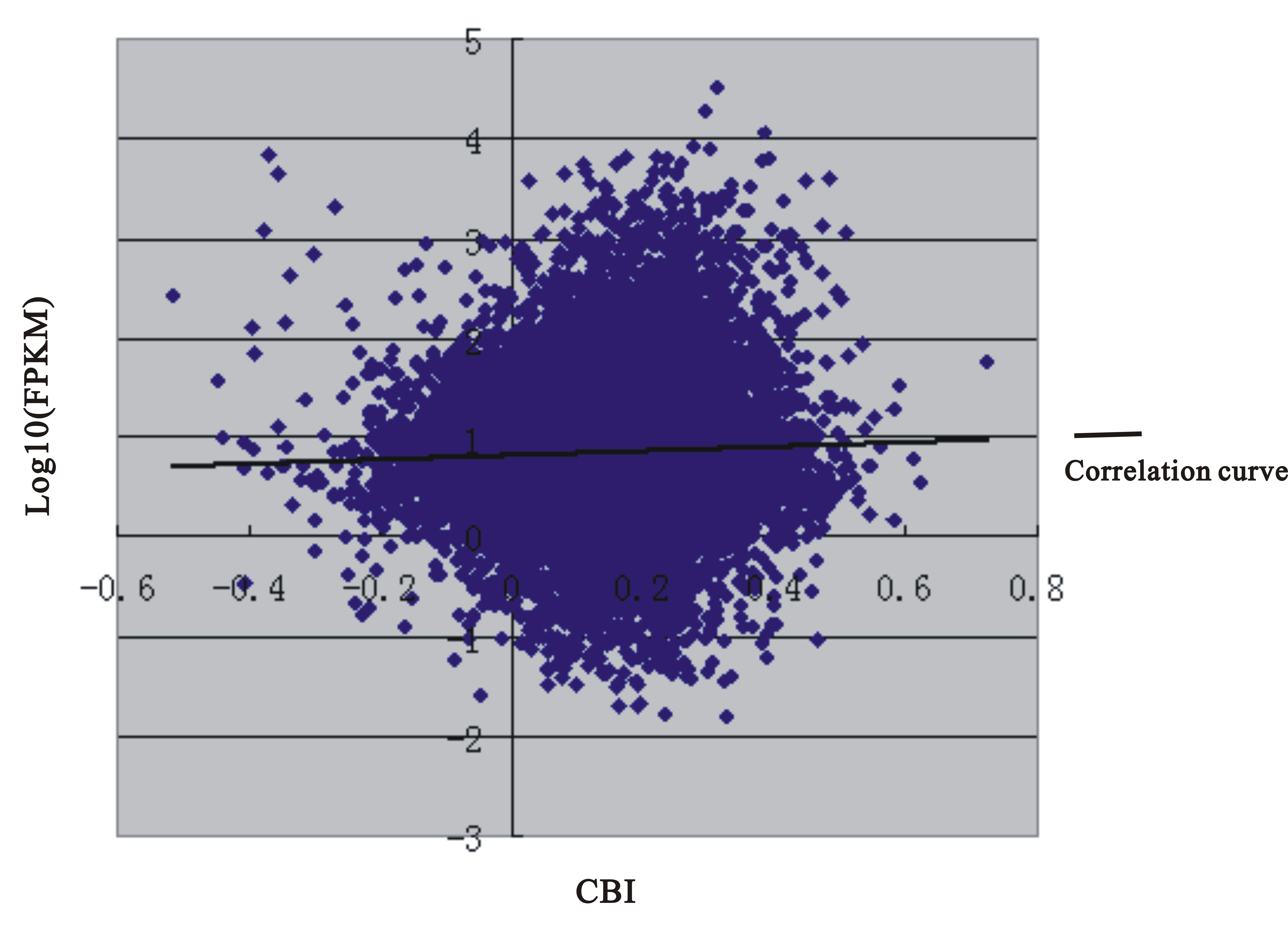

Supplement: Additional file 6 — Relationship between CBI (codon bias index) and expression level (Log10 (FPKM)) of all An. sinensis transcriptome unigenes. [file 1756-3305-7-314-S6.jpeg]
