# Supplementary material for: De novo transcriptome sequencing and sequence analysis of the malaria vector Anopheles sinensis (Diptera: Culicidae)
Source: Parasit Vectors. 2014 Jul 7;7:314. doi: 10.1186/1756-3305-7-314 (PMC4105132; doi:10.1186/1756-3305-7-314)
Supplement: Additional file 3 — Total codon usage and codon usage bias in An. sinensis transcriptome. [file 1756-3305-7-314-S3.doc]

**Additional file 3 Total codon usage and codon usage bias in *An. sinensis* transcriptome.** A total of 4,048,4587 codons in 24,361 ORFs of *An. sinensis* transcriptome were used in the calculation of codon usage bias measured by RSCU.

| **Codon** | **Amino Acid** | **Total codon counts** | **RSCU** | **Codon** | **Amino Acid** | **Total codon counts** | **RSCU** |
| --- | --- | --- | --- | --- | --- | --- | --- |
| **CUG** | **L** | 150300 | **2.503** | **GAA** | **E** | 110359 | 0.845 |
| **CCG** | **P** | 114590 | **1.960** | **CCA** | **P** | 50482 | 0.807 |
| **UCG** | **S** | 99624 | **1.857** | **AUU** | **I** | 51414 | 0.770 |
| **CGC** | **R** | 68059 | **1.725** | **GCA** | **A** | 54217 | 0.753 |
| **GUG** | **V** | 108847 | **1.693** | **CCC** | **P** | 44102 | 0.753 |
| **ACG** | **T** | 105324 | **1.680** | **AGU** | **S** | 39887 | 0.744 |
| **AUC** | **I** | 114650 | **1.675** | **AAA** | **K** | 80576 | 0.706 |
| **CGG** | **R** | 57312 | 1.509 | **GUU** | **V** | 42748 | 0.703 |
| **GCC** | **A** | 114098 | 1.444 | **CUU** | **L** | 35938 | 0.661 |
| **AGC** | **S** | 75475 | 1.400 | **CAU** | **H** | 40079 | 0.644 |
| **UAC** | **Y** | 93787 | 1.372 | **UGG** | **W** | 37268 | 0.610 |
| **GGC** | **G** | 93641 | 1.346 | **CAA** | **Q** | 60076 | 0.608 |
| **AAC** | **N** | 129735 | 1.341 | **AAU** | **N** | 57231 | 0.607 |
| **CAG** | **Q** | 136649 | 1.328 | **UUU** | **F** | 43719 | 0.599 |
| **ACC** | **T** | 84459 | 1.321 | **GGG** | **G** | 36540 | 0.572 |
| **UUC** | **F** | 96902 | 1.253 | **ACA** | **T** | 35433 | 0.570 |
| **GCG** | **A** | 92718 | 1.224 | **GCU** | **A** | 42635 | 0.554 |
| **AAG** | **K** | 149125 | 1.217 | **CUA** | **L** | 28767 | 0.524 |
| **CAC** | **H** | 69747 | 1.148 | **GUA** | **V** | 30822 | 0.515 |
| **UCC** | **S** | 61796 | 1.145 | **UGU** | **C** | 23441 | 0.511 |
| **CUC** | **L** | 65739 | 1.131 | **UCA** | **S** | 25568 | 0.471 |
| **GAG** | **E** | 150294 | 1.094 | **AUA** | **I** | 28647 | 0.462 |
| **GGU** | **G** | 79894 | 1.076 | **UAU** | **Y** | 28762 | **0.444** |
| **GUC** | **V** | 66977 | 1.039 | **CCU** | **P** | 23202 | **0.385** |
| **GAC** | **D** | 117552 | 1.038 | **ACU** | **T** | 24129 | **0.383** |
| **CGU** | **R** | 39418 | 1.015 | **UCU** | **S** | 18892 | **0.356** |
| **UGC** | **C** | 44741 | 0.968 | **AGA** | **R** | 12220 | **0.351** |
| **GGA** | **G** | 64104 | 0.945 | **AGG** | **R** | 12430 | **0.342** |
| **CGA** | **R** | 32694 | 0.909 | **UUA** | **L** | 14567 | **0.284** |
| **GAU** | **D** | 98370 | 0.902 | **UGA** | ***** | 551 | 0.025 |
| **AUG** | **M** | 88870 | 0.880 | **UAA** | ***** | 312 | 0.017 |
| **UUG** | **L** | 47679 | 0.851 | **UAG** | ***** | 274 | 0.017 |
